# Roadmap for a Participatory Research–Practice Partnership to Implement Evidence

**DOI:** 10.1111/j.1741-6787.2012.00256.x

**Published:** 2012-11

**Authors:** Margaret B Harrison, Ian D Graham

**Affiliations:** Professor, School of Nursing, Scientific Director of the Practice and Research In Nursing Group, Queen’s UniversityKingston, Ontario, Canada; Associate Professor, School of Nursing, Queen’s UniversityKingston, Ontario, Canada.

**Keywords:** evidence-based practice, activating knowledge, planned-action framework, engaged scholarship, action-research

## Abstract

**Background:** Our research team has undertaken implementation of evidence in the form of practice guideline recommendations for populations in hospital, community, and long-term care settings with diverse provider and patient populations (people with chronic wounds, e.g., pressure and leg ulcers, heart failure, stroke, diabetes, palliative care, cancer, and maternity care). Translating evidence into clinical practice at the point of care is a complex and often overwhelming challenge for the health system as well as for individual practitioners.

**Purpose:** To ensure that best available evidence is integrated into practice, “local evidence” needs to be generated and this process accomplishes a number of things: it focuses all involved on the “same page,” identifies important facilitating factors as well as barriers, provides empirical support for planning, and in itself is a key aspect of implementation. In doing this work, we developed a roadmap, the Queen’s University Research Roadmap for Knowledge Implementation (QuRKI) that outlines three major phases of linked research and implementation activity: (1) issue identification/clarification; (2) solution building; and (3) implementation, evaluation, and nurturing the change.

In this paper, we describe our practical experience as researchers working at point-of-care and how research can be used to facilitate the implementation of evidence. An exemplar is used to illustrate the fluid interplay of research and implementation activities and present the range of supporting research.

**Implications:** QuRKI serves as a guide for researchers in the formation of a strategic alliance with the practice community for undertaking evidence-informed reorganization of care. Using this collaborative approach, researchers play an integral role in focusing on, and using evidence during all discussions. We welcome further evaluation of its usefulness in the field.

## INTRODUCTION

Care improvement guided by external evidence and local circumstances has been a longstanding focus with issues that arise at the point-of-care (Harrison et al. 1993). One underlying premise of this focus is that health care based on “best available evidence” occurs with the adoption and implementation of practice guidelines. Indeed, when delivered appropriately, guidelines have the potential to aid in consistent, high-quality care delivery resulting in superior health outcomes (Grimshaw & Russell 1993; Grimshaw et al. 2003).

Since the mid-1990s, the proliferation of guidelines from credible bodies such as the Royal College of Nursing, Agency Health Care Policy and Research, Scottish Intercollegiate Guidelines Network and others, has provided impetus for care settings to focus on care delivery supported by best available evidence and the use of guidelines. As knowledge tools, practice guidelines represent a major advance in transferring research evidence from a multitude of studies for use at the point of care. Best available evidence is now more accessible and packaged in a more useable form. Guidelines also serve as a vehicle to improve consistency in the structure and process of care both within and across settings thus providing organizations with a “script” as they undertake efforts to deliver evidence-driven care.

On the surface, this seems straightforward: by translating available evidence into practice recommendations, then integrating practice recommendations into service delivery, the result will be improved quality of care and health outcomes. The underlying assumption to this sequence of events is that, when good evidence is embedded in a quality guideline, it will be fairly straightforward to move it into practice.

In reality, however, translating evidence into clinical practice at the point of care is a complex, often overwhelming challenge. In one such effort, our large teaching hospital made a strong commitment to implement guideline recommendations to improve pressure ulcer care. Nonetheless, it took many years of concerted effort to achieve modest gains (Fisher et al. 1996; Harrison et al. 1996; Harrison et al. 1998; Harrison et al. 2006).

Further to field efforts, such as ours, focused on guideline uptake, numerous models, and frameworks for evidence-based practice emerged to guide implementation. Ciliska and colleagues examined eight models to guide implementation. Overall they noted a commonality of steps and a recognition of “the need for a systematic approach to practice change” (Mazurek Melnyk & Fineout-Overholt 2010, p. 272). In another synthesis of selected models, Rycroft-Malone and Bucknell (2010) critically examined key dimensions postulated to be related to robustness and application. Their analysis provides a means to assess and select a model/framework for specific practice projects. Less clear from these scholarly compilations is how these models/frameworks involve research activity (if at all) and how that might facilitate implementation.

Guideline proponents have recognized that practice recommendations serve only as the first step of a much larger effort (Toman & Harrison 2001). Rycroft-Malone et al. (2004) describe evidence as being comprised of research, clinical experience, patient and caregiver experience, and local context information. This broad understanding of evidence provides insight into the crux of the issue, that is, the important local work required first to capture all the “evidence,” not simply the use of the external research housed in guidelines or other knowledge tools. This dynamic evidence changes at the bedside is poorly understood. This paper is focused on the engaged research in acquiring this broad evidence as an active, and proactive, element of implementation. Having worked through implementation processes with different groups, we recognize two important aspects: the iterative nature and linkage between local implementation activities, and the possibility to generate local evidence through “planned action” research. Both can be greatly assisted by a research–practice partnership.

Based on our experiences, we offer a roadmap for researchers to engage in a collaborative research–practice approach to implementation. The approach is illustrated using community wound care as an exemplar (see Table 1) and describes the types of research undertaken to support implementation. We hope this roadmap will provide guidance on how to plan, execute, and evaluate implementation both for researcher-practice partners and for practice settings without researcher–partners.

**Table 1:** Wound care exemplar

Leg ulcers and their treatment have been remarkably well documented for centuries. A cathedral window in York, UK (12th & 13th centuries AD) depicts a practitioner treating the lowly leg ulcer. Much later, Florence Nightingale noted that more than half the working class men in London suffered from this condition (Baly 1997). Remedies available to early practitioners were primitive yet, one consistent, conservative approach to managing these wounds emerged—the use of compression. Slowly compression bandaging, and the many ways it was applied, underwent scrutiny and evaluation, ultimately producing a body of evidence for its effectiveness in venous disease. This culminated with a conclusive Cochrane review in 1999 (Cullum et al. 1999) that provided clear, unequivocal evidence for conservative management of venous leg ulcers: (1) assessment: performance of a comprehensive clinical assessment including an Ankle–Brachial-Pressure-Index to determine nature of the ulcer (venous, arterial, or mixed etiology); and (2) management for healing: use of high compression bandages.

## ORIGINS OF THE APPROACH WITH PARTICIPATORY RESEARCH

For more than 15 years our interdisciplinary research team has undertaken implementation of evidence in hospital, community, and long-term care settings with diverse provider and patient populations, for example, chronic wounds (pressure and leg ulcers), heart failure, stroke, diabetes, palliative care, cancer, and maternity care. To ensure that best available evidence will actually be integrated into practice, we found that research engagement was required to generate local evidence about the implementation context. The meaning of research in this circumstance refers to local studies that employ scientific methods vs. generalizable “capital R” research.

Conducting research to generate local evidence accomplishes a number of things: it focuses all involved on the “same page,” identifies important facilitating factors and barriers, provides empirical support for planning, and is, in itself, instrumental to implement and sustain change.

### Research–Practice Partnerships

The research–practice partnership is an alliance that includes clinical and service level decision-makers, recipients of care, healthcare professionals, and researchers. Our approach to evidence-informed practice begins when a clinical setting identifies a care issue. The decision-making process is iterative and interactive, incorporating external evidence from the literature and local planning. As researchers, we assist groups to move incrementally and strategically toward the goal of an evidence-informed service. First, an enquiry about the local context and population is necessary to enable effective uptake. This type of field research is referred to as “integrated knowledge translation”—an active participatory collaboration with end-users working together to shape the research process within a specific context (see http://www.cihr-irsc.gc.ca/e/26574.html). Researchers and end-users collaborate to define and refine the questions, decide on the methodology, collect data and develop tools, interpret findings, and finally, implement findings and disseminate the research results. Gibbons et al. (1994) refers to the approach that deals with specific issues and problems from the real world to generate socially relevant research as “Mode 2” knowledge production, that is, where science is problem-focused and context-driven. Traditional research (labeled as “Mode 1”), is characterized as academic and investigator-initiated.

### A Planned Action Approach

**Figure 1:**
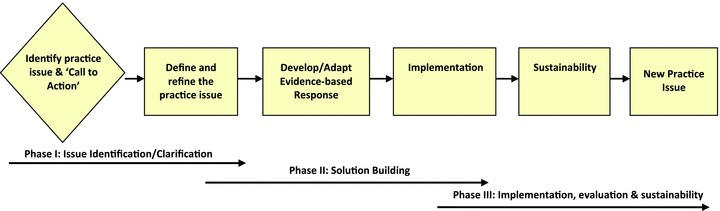
Queen’s University research roadmap for knowledge implementation.

The Queen’s University Research Roadmap for Knowledge Implementation (QuRKI) makes explicit the mutually supporting and interconnected cycles of research studies supporting implementation. Studies are characterized by the use of multiple and mixed research methods. There is a logical flow of enquiry, but it is neither linear nor tightly sequential. External evidence (from the literature) serves as foundation providing leverage in (re)designing the service by discovering how to “fit” and “align” this evidence with local resources, populations and context. QuRKI consists of three major phases of linked research and implementation activity (see Figure 1):

1. Phase I: Issue Identification/Clarification.
2. Phase II: Solution Building.
3. Phase III: Implementation, Evaluation, and Nurturing the Change.

Working closely with practice and health services partners is key to this approach. Studies are pragmatically designed and conducted at each phase to provide timely decision-making information. At each phase the accompanying research that contributed to a successful implementation is illustrated.

### Phase I: Clarification

#### Issue identification

Issue identification by the practice group within its local context, rather than by researchers, is crucial. Enquiry is driven from a practice issue rather than researcher curiosity. Local experience and expertise raises awareness of the issue providing the momentum for a strategic alliance of players. Thus, Phase 1 is characterized by the formation of a strategic alliance among providers within or across agencies and settings with the researchers. Ideally, the alliance will have clinical and management expertise as well as representation from an appropriate range of decision-making levels.

During Phase 1, typical questions that may require research include:

- What is the issue? Who is concerned?
- What is the relevant population of patients and providers?
- What is the extent/magnitude of the issue?
- What does the external evidence suggest is the best practice for care of this population?
- How similar is the local population to those reported in published research studies?

Some questions may be answered internally by the organization through their available administrative databases, such as patient rosters to determine the number of people with the condition on service. Others questions may require time and expertise unavailable within the setting itself. Partnering with researchers provides the expertise required if there is a need to gather primary data through a local study. Methods could include prevalence, incidence and population profiling studies, and environmental scans.

To begin, a critical review and quality assessment of the external evidence and/or practice guidelines should be undertaken to ascertain best available evidence related to the care issue. Then, an assessment conducted locally will determine the extent of the issue, the environment in which it occurs, and how care is currently delivered. Answering these questions develops the foundation for decision making and planning for any changes. The purpose of Phase 1 is to create a reliable, comprehensive and *shared* understanding of the issue and care needed to address it.

#### Wound care exemplar: Getting serious about organizing for evidence-based care

In 2000, our local homecare authority, serving an urban–rural population of ∼1,000,000 people, was concerned that expenditures for the leg ulcer population were disproportionate to other healthcare populations served. Nursing time was becoming an increasingly scarce resource, with provision of leg ulcer care seen as a troublesome area from cost and resource perspectives. We were asked to join forces with the homecare authority and a community-nursing agency (Graham et al. 2006) to tackle the issue. Both the researchers and practice stakeholders were interested in evidence-based practice and implementation for care improvement. During this call-to-action, a strategic alliance was formed to explore the issue and understand resource implications.

**Figure 2:**
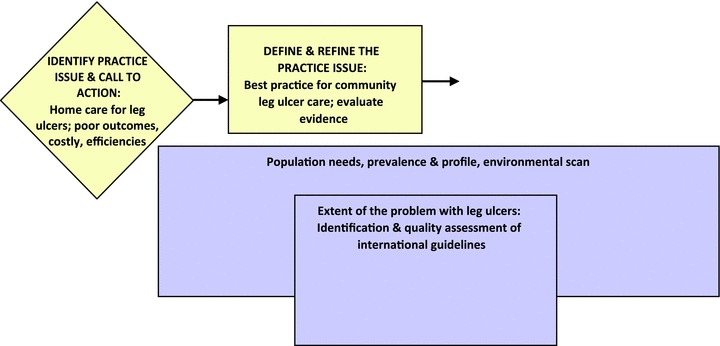
Phase I: Issue/problem identification/clarification.

Although questions around expenditures for the community-dwelling wound populations drove the first enquiries, even more questions arose when the administrative databases revealed high visit frequency and a vast array of bandages and dressings used. By engaging frontline practitioners and managers in issue clarification (Figure 2) questions were collaboratively identified and refined resulting in a number of enquiries, including observational and integrative studies to understand the local population and context and to identify available evidence for leg ulcer care.

#### Prevalence and profile studies

Mixed methods were used to produce local evidence about the issue and the affected population. Because we were unable to determine the size and profile of the population from administrative databases, we undertook a regional prevalence/profile study to assess if our local population was similar to those reported in the literature and whether external evidence was applicable. Importantly the profile of the population revealed an age distribution and mobility status that would allow the use of clinic care in addition to home-based care as a delivery option. Given the longstanding nature of leg ulcers and high recurrence rates, even incremental improvements in management were worth considering. Lastly, the population size represented an economy of scale where it was postulated that one nursing agency specialized in wounds would be more efficient than the current multiple agencies.

#### Integrative studies and quality assessment of international guidelines

When the leg ulcer initiative began in 2000, guidelines were available internationally but there were no Canadian-specific guidelines. Facing the challenge of importing guidelines from another country and adapting these recommendations for local use, we first conducted an analysis of available international recommendations (Graham & Logan 2004; Graham & Harrison 2005) using an interdisciplinary approach with community nurses, family, and specialist physicians. The process was educative for all involved and provided the mechanism for nurses and physicians to recognize there was solid evidence available for effective care. This evidence served as external leverage and a seed for change. Because our local population was similar to those in the internationally reported data (e.g., rate of leg ulcer occurrence, recurrence, profile of the group) there was confidence about using the international recommendations. This important information provided a firm basis toward moving to Phase II, Solution Building.

### Phase II: Solution Building

In the Solution Building phase, the research–practice alliance works with the local information focusing on what implementation of a “best practice” innovation would entail in their context. This is a proactive exchange to identify changes in the current practice and delivery of care.

Solution building questions might include:

- What is the gap between current practice and the recommended practice?
- How appropriate and acceptable are the best practice recommendations for delivery in this context? How does the guideline(s) need to be adapted before implementation?
- What would be needed to organize (or re-organize) practice and health services to deliver care as recommended by the guideline?
- What are the supporting factors and the barriers related to implementing the best practice, the adopters, and the care environment?

Research during this phase is population-based and may include: environmental scans to understand the current organization and delivery of care; knowledge, attitudes, and practice (KAP) surveys with local providers; gap analyses through practice audits; and barriers assessment to determine what might help or impede a reorganization based on external evidence. Some factors may be known but others are more difficult to tease out. This process provided the insights needed for key players to identify clinical, health services, and/or policy changes (small or large) to move forward with evidence-informed care.

#### Wound care exemplar: What will we need to do to change things?

**Figure 3:**
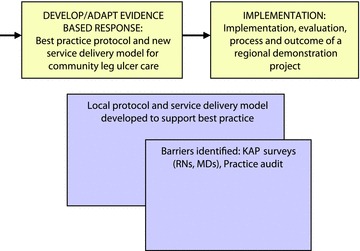
Phase II: Solution building.

Having clarified the issue and described our local wound population characteristics, our next step was to discover the gap between current and recommended practices. International leg ulcer guideline recommendations provided the basis to develop a local practice protocol. We strove to understand the barriers and facilitators locally that would enhance or deter the reorganization of homecare delivery for optimal service to leg ulcer patients. Several research enquiries contributed to this (Figure 3).

#### Gap analysis

The lead nurse in the leg ulcer service conducted a gap analysis as part of her graduate research (Lorimer et al. 2003). Data revealed a lack of attention to a key element with leg ulcer management namely, specification of etiology. On referral to homecare, only half the cases had documented etiology and, after referral, ankle–brachial pressure index (ABPI) measurements were not performed consistently. Furthermore, many care providers lacked the equipment (portable ultrasound dopplers), the knowledge (best practices), the skill (perform Doppler assessments), or the training (application of bandaging technologies). A disquieting finding showed that only 40% of people with venous leg ulcers were being treated with compression bandages, the best practice recommendation (Lorimer et al. 2003). These gaps in assessment and management practices became the focus for change.

#### Knowledge, attitudes, and practice surveys (KAP)

Important findings emerged through community nurse and family physician surveys (Graham 2001; Graham et al. 2003). Although results showed significant gaps in nurses’ knowledge about the evidence for effective venous leg ulcer care, even larger gaps in knowledge were revealed among family physicians responsible for ordering care (Graham et al. 2000a). However, importantly for implementation, both nurses and physicians had positive attitudes about leg ulcer care and expressed a strong willingness to use a standard, evidence-based protocol for care.

#### Environmental scan

The environmental scan, including assessment of expenditures, provided powerful baseline and planning information about the current state of affairs in the region and where changes would be required. Over an average 4-week period, the scan revealed that each client was treated by an average of 19 different nurses and of these, 40% received visits daily or twice a day. Projected annual cost, based on 192 cases per 4-week period, was $1.26 million in nursing and supply expenditures (Friedberg & Harrison 2002). However, if recommended best practices were instituted with compression bandages applied by skilled practitioners, visits would be reduced to an average of 1–2 visits weekly, potentially saving visits and reducing costs. This information proved to be decisive in fuelling change.

#### Health services and delivery models analyses

To determine how best to reorganize service provision to conform to best practice in the most economical way possible, the research–practice partnership analyzed available research that evaluated wound care delivery modalities, in particular clinic and homecare service models. In our region homecare is delivered by numerous geographically organized nursing agencies contracted for wound care services. All nurses in any agency delivered care to multiple health populations, including chronic wound care. Agency remuneration did not support the time required to conduct an intensive evidence-informed comprehensive assessment (∼1.5 hours) before initiating compression technologies, and changing this policy to allow more time on the first nursing visit was not considered feasible at the system-level. However, using the synthesis of health services research to illustrate how other settings organized and set-up delivery models, we demonstrated how a typical episode of leg ulcer care might be managed locally. Then, practitioners, decision makers, and planners, facilitated by the researchers and the research data, designed a new regional service wound-focused delivery model.

A pressing knowledge gap identified the need for more specialized training and ongoing professional practice support for the RN-team. An educational program through a UK university provided master classes at a local vascular clinic for nurses appointed to the wound team. A train-the-trainer model was then adopted for ongoing education.

In summary, the local community nurses and homecare managers worked to develop a model supporting evidence-informed care. At this juncture the agencies took advantage of the opportunity to explore the benefits and disadvantages of clinic care electing to evaluate both clinic and in-home care options through a demonstration project. Importantly, no commitments to permanent changes had to be made by the region until the evidence of the effect of the change was produced.

### Phase III: Implementation, Evaluation, and Nurturing the Change

**Figure 4:**
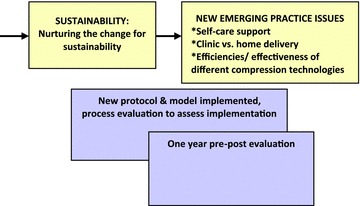
Phase III: Implementation, evaluation and sustainability.

Phase III (Figure 4), focuses on the actual implementation of the guideline and an evaluation of its uptake and outcomes. Planning for implementation underpins both Phases I and II, and culminates in Phase III, where a process evaluation revealed important process-of-care information about delivering evidence-informed recommendations (e.g., organization of teams to carry-out the comprehensive assessment). As well, experience with the feasibility, appropriateness, and sensitivity of outcomes may later be useful for quality monitoring. Nurturing change requires proactive work to promote sustainability.

Questions requiring a research approach at this stage include:

- What will indicate that we have implemented the new care protocol?
- What (new) indicators and processes could be embedded in quality monitoring with the implementation of guideline recommendations?
- How effective and efficient was the reorganization of care locally in adhering to the guideline recommendations?
- What effect has the reorganization for evidence-based care and use of the recommendations, had on patient, provider, and system outcomes?
- How will the change be sustained over time?

The process evaluation can range from a simple audit to a more comprehensive, formal program evaluation of the new approach to care. The input of the providers and decision makers at this stage is instrumental to identify whether or not quality of care processes or indicators appropriate for the process evaluation are already in place. Using “what exists” streamlines data collection and augments quality and research efforts. If shortfalls are noted then additional implementation strategies can be introduced. As a formative endeavor, the process oscillates between implementation and evaluation. Program evaluation designs that include process-of-care elements may be used to pilot approaches for future use, for example, audits for specific assessment recommendations. Full-scale evaluations examine individual, provider, and/or system outcomes/impact (e.g., healing, nurse satisfaction with professional practice, emergency visits, or readmissions for poor management) using the most feasible, rigorous design (e.g., pre-post cohort study, health services randomized controlled trial, etc.). Researchers must consider issues such as the pros and cons of longitudinal versus cross-sectional data collection, using primary data, and/or data from administrative databases and, of course, how the evaluation will be funded (Graham et al. 2000b; Bick & Graham 2010; Godfrey et al. 2010; Graham et al. 2010).

In Phase III, the alliance begins to understand the impact and benefit of their work by conducting process and outcome evaluations. This phase presents opportunity to assess potential longer term monitoring and outcome assessment for the setting.

#### Wound care exemplar: Were we successful implementing and did we make a difference?

Working closely with our practice and health services partners, all phases were pragmatically designed to provide data in a timely fashion for planning and decision making. The implementation strategies to deliver evidence-informed care included: A local protocol with one-page guide, documentation tool embedding the evidence-based assessment, lead nurse (combination CNS/manager), an all-RN wound care team, support and education for the team along with commitment to evaluate the changes. To determine our success, implementation was assessed at both the practice and health services levels.

A demonstration project was conducted to assess the formative and summative evaluation components of the implementation research. Demonstration projects are time-limited, considered an “experiment” followed by a decision at the completion of the evaluation. Nonetheless, though the formative evaluation is intended to improve the implementation process, ultimately it is the decision makers who will gauge the value of sustaining the implementation.

#### Wound exemplar regional demonstration project: Did it make a difference?

During this research cycle, a 1-year regional demonstration project was launched (Figure 3). The regional authority reserved commitment to the changes until evaluation of the effectiveness and efficiency of the new approach was known. Using a program evaluation design and quality indicators, we conducted a process evaluation to ensure that the new protocol and delivery system were actually in place. Our community partners designated the benchmarks and process elements to be scrutinized. For instance, clinic care would be considered up-and-running effectively if half-day appointment blocks were filled at the two clinic sites and individuals were seen within 15 minutes of their appointment time. The completeness, quality, and timeliness of assessments were determined through quality audits.

#### Pre-post process and impact evaluation

Once the benchmarks and process elements were satisfied and after a wash-out period between old and new service delivery models, we undertook a 1-year pre-post evaluation to measure patient and system outcomes (Harrison et al. 2005). The same team provided care in both home and clinic settings using an evidence-informed protocol.

Before implementation of the leg ulcer service, the 3-month healing rate was 23%, which improved to 56% in the year following the implementation of the evidence-based care. During the same period, nursing visits decreased significantly (median of 3 to 2.1 per week) as did daily visits (38% to 6%). Supply costs were significantly less (median of $1,923 per episode of care to $406 per episode). In this case, evidence-based practice was not only more effective but less expensive than usual care. Individuals were being healed more quickly, thus the home care authority was able to care for more people using the same resources. Continuity of care improved with the new delivery model, adding to satisfaction with professional practice.

Over time process-of-care tools evolved to assist individuals with their leg ulcer self-care and management, for example, self-care education was built into a section of the care plan and patient handouts developed. A local dermatologist and vascular specialist both identified efficiencies in quicker referrals of more serious cases highlighting the value of home-based screening by nurses as a time-saver for their specialist practices.

**Figure 5:**
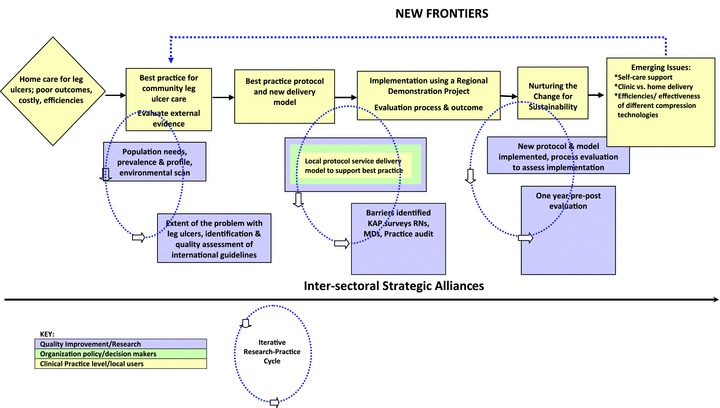
Queen’s University research roadmap for knowledge implementation illustrated with leg ulcer exemplar.

As one may surmise, the progression was not quite as straightforward as the tidy silos presented here imply. Figure 5 portrays the continuous, iterative interplay between implementation and research activity. As an “alliance for change,” practitioners and decision makers had to be equally dedicated to reorganizing structures and processes to support delivery of evidence-informed practice. On occasion as researchers, we found ourselves negotiating changes as informed, third-party players. For example, to free up enough assessment time within the visiting roster, we argued for extended visits with the homecare authority on behalf of the nursing agency that would lose income in carrying this out. In another instance, formal research and the contract with the national funding agency actually influenced the continuance of the initiative when significant cut-backs were underway.

#### Sustainability and new issues

Several key elements contributed to the ongoing delivery of evidence-informed care. First, the active participation of the nurse leader of the clinic and home wound service was pivotal. She interacted as mentor, consultant, and educator not only for her team but for ongoing advancement of wound care regionally with other home nursing agencies and, from a clinical management perspective, she was instrumental in embedding the evidence in normal processes of care. For example, in creating the Assessment Forms, her design was structured so that venous elements fell on one side of the page and arterial ones fell on the other, permitting clear assessment of wound etiology.

A second sustaining characteristic is development of practice quality indicators drawn from the process evaluation. Using the key outcome assessment of healing at 3 months, all unhealed cases at that time-point were reviewed by the team and the plan-of-care evaluated and modified as necessary. This aspect of embedding the evidence in everyday process should be a focus. The sustainability elements were informed by the key guideline recommendations once operationalized and refined by the local research-implementation efforts.

There is a postscript to the wound care story that brings it full circle. Each solution engenders new questions. During the Demonstration Project and the 1-year pre-post study, attention had turned to sustainability, with new questions coming to light as the cycle progressed. Two examples are highlighted:

First, clinic versus home. The home care authority and nursing agencies were convinced of the merit of the evidence-based approach, but they wondered whether application of the protocol would be equally successful if rigorously evaluated in both nurse clinic and in-home care settings. This question was subjected to a randomized controlled trial within the 1-year post period of the evaluation study (Harrison et al. 2005). The organization of care, not the setting where care is delivered, influences healing rates. Key factors were identified such as a system that supports delivery of evidence-based recommendations and care being provided by a trained nursing team. The RCT reassured care agencies that they can expect similar outcomes in terms of healing quality of life and pain thus they could continue to offer services in either setting. Patient preference in selecting their venue of care was also identified in this trial and a cohort study was subsequently conducted (Harrison et al. 2011b).

Second, high-compression bandaging systems. Questions arose about the provision of various high-compression bandaging technologies having different nursing time, cost, and supply implications. We undertook a technology trial based on our previous experience working with the providers and decision makers, where the sample size calculation was driven by administratively important outcomes. The trial’s primary outcome was a difference of 4-weeks to healing during an episode of leg ulcer care. This was the “break-even” point where home care authorities might consider using one system over another as first-line therapy. This RCT is now completed and is one of the largest Canadian wound trials (>420 patients, 10 sites) (Harrison et al. 2011a).

Perhaps the best indication of sustainability is that years later the wound service continues in the region and the approach has extended to chronic wounds generally. Care is still available in both clinic and the home. Our collaboration expanded over time to include healthcare authorities in three different provinces as we progressed with implementation and began producing primary evidence in large-scale studies.

## CONCLUSIONS

Effective solutions for longstanding, complex healthcare issues are possible through collaborative research focused on promoting evidence uptake. Adapting external evidence to align and tailor it to the local context can be aided by a participatory approach between a practice group and researcher(s). It is engaged scholarship (Gibbons et al. 1994) at the point-of-care responding to field issues/concerns in moving evidence into day-to-day-practice.

This approach to research and implementation is more complex than solely investigator-initiated research. Our wound exemplar demonstrates the spectrum of collaborative research used to achieve evidence-informed implementation and maximum “buy in” at the practice level. The effort resulted in more effective, less expense care; the quality of care provided in the region improved substantially, and a greater number of individuals are cared for with the same allocation of home care dollars. Importantly, scarce nursing resources are used more efficiently.

Practitioners, policy makers, and researchers built on local knowledge and evidence to bring external evidence to bear. The Queen’s Roadmap (Figure 5) lays out this iterative process of using research to facilitate the implementation of evidence using an action-research model to produce local evidence. Research activity illustrates a continuous cycle of producing and using evidence. Implementation activity with practitioners and decision makers is highlighted in the yellow boxes, whereas the blue boxes represent contributing research elements. In working with groups planning for evidence-informed services, understanding the major supports and barriers at practice, health services, population, and decision-making levels is a key enabling factor requiring a proactive, incremental, and iterative technique of threading research where appropriate. Lessons learned include:

1. A strategic and collaborative alliance between researchers and health settings is an essential starting point for this type of partnership for change in quality approaches.
2. Graduate students and those in the field learned Mode 2 science (i.e., working with end-users) and its strengths naturally by participating.
3. The implementation process is not linear or sequential but typically simultaneous and iterative.
4. Attending to both the clinical/practice and health services levels concurrently is vital; typically system changes are of great consequence.
5. Responsiveness to decision makers and their challenges is essential to maintain an alliance for change.
6. Careful balance between methodological rigour, feasibility, and timeliness is needed.
7. All parties must be committed to sound population-based health principles and needs-based planning.

We developed the Queen’s Roadmap based on real results achieved through using a collaborative research–practice approach to reorganization. The role of research is a means to an end—where the end is improved care through evidence-informed practice. As researchers we help create the strategic alliance and solutions-focused collaboration, and play an important educational function in defining and redefining research questions and methodological approaches required to obtain answers. The research team needed the intellectual flexibility to use a full toolkit of methodologies balancing rigor, timeliness, and feasibility. In this way, researchers facilitate change through the use of best available methods for each step (organizational planning, guideline appraisal and adoption, evaluation of implementation). For example, to understand local populations one first turns to available administrative datasets to develop a profile and, only if necessary, conduct primary research. Or from a research perspective it may be preferable to design a cluster randomized trial to evaluate a guideline implementation, but if decision makers are planning a regional reorganization for the next fiscal year, a pre-post study may be the most workable solution to provide data. We found that ongoing research for additional questions aided in sustaining the alliance.

From the practice setting, clinicians, managers, and policymakers provide information on what matters to them in the day-to-day world and offered a reality check in terms of feasibility, realistic targets, and what was possible. They were open to accessing, collecting, and using local data for planning and decision making. This was an essential ingredient in formulating a quest for data to define the new reorganization and what it would take including decision-making aspects, setting priorities, communicating the vision and priorities within the setting. The opportunity to work with practitioners and decision makers willing to invest the time and resources into solution-building is an extraordinary one. Even though people in the roles changed, the alliance sustained to see the process through over a number of years ensuring that each step of the journey led to discovering a path for better outcomes.

This collaborative approach allows researchers to play an integral role in focusing on external and local evidence during all discussions. We would welcome further field evaluation of the Queen’s Roadmap’s usefulness.
